# The Effects of Progesterone on Glial Cell Line-derived Neurotrophic Factor Secretion from C6 Glioma Cells

**Published:** 2012

**Authors:** Parichehr Hassanzadeh, Elham Arbabi

**Affiliations:** 1 *Research Centre for Gastroenterology and Liver Diseases, Shahid Beheshti University of Medical Sciences, Tehran, Iran*

**Keywords:** C6 glioma cells, GDNF, Progesterone

## Abstract

**Objective(s):**

Progesterone is a steroid hormone whose biology has been greatly studied within the confines of reproductive function. In recent years, the neuroprotective effects of progesterone have attracted growing interest. Glial cell line-derived neurotrophic factor (GDNF), is a neurotrophic factor which plays a crucial role in the development and maintenance of distinct sets of central and peripheral neurons. In the present study, we investigated the potential implication of GDNF in the neuroprotective action of progesterone.

**Materials and Methods:**

Cultured rat C6 glioma cells were treated with progesterone (100 nm, 1 µM, and 10 µM) or its vehicle. After 24, 36, 48 and 72 hr, GDNF protein levels were measured in the cell-conditioned media and cell lysates using a GDNF ELISA kit. Cell numbers were determined by a cell-counting assay kit.

**Results:**

Forty-eight hr treatment with progesterone (10 µM) resulted in a significant elevation of GDNF secretion from C6 glioma cells that remained elevated up to 72 hr. The intracellular content of GDNF and cell numbers were not affected by progesterone treatment.

**Conclusion:**

Stimulation of GDNF release from glial cells appears as a potential mechanism through which progesterone exerts its neuroprotective effects.

## Introduction

The female sex steroid, progesterone, is a major gonadal hormone which mainly contributes to the protection of fetus during the gestation period (1). Progesterone, besides its effects on the reproductive and endocrine systems, acts as a neurosteroid in the central nervous system (2-4) and exerts a wide range of actions depending on the target tissue (5-7). This neuroactive steroid has been found to be beneficial as a neuroprotectant in a number of animal species and in several modes of neurological injuries (8, 9). In fact, observing a sex difference in response to an experimentally induced traumatic brain injury was the first evidence for the neuroprotective properties of progesterone. According to Stein, females tend to recover more quickly from traumatic brain injury that may have a hormonal basis (10). He found out that experimentally brain-injured female rats with elevated levels of serum progesterone sustain less neurological damage and recover better than female rats with low progesterone levels at the time of injury. In the peripheral neuropathies, progesterone has been shown to promote the remyelination and axonal regeneration (11). Furthermore, treatment with progesterone restores the expression of molecular markers that characterize motoneurons and promotes proliferation and differentiation of oligodendrocyte progenitors in the experimental spinal cord injury (12, 13). The protective effects of progesterone following cerebral ischemia have also been shown (14, 15). Meanwhile, the mechanism(s) through which progesterone exerts its neuroprotective effects are not clearly defined. There are reports indicating that progesterone protects cultured PC12 cells against the death due to the deprivation of neurotrophic support (16, 17). Neurotrophic factors are proteins that exert survival-promoting and trophic actions on neurons in the peripheral and central nervous systems (18, 19)*. *In recent years, a growing interest has been attracted towards the protective effects of neurotrophic factors in various types of neuronal pathologies (20-23). In this context, an accumulating body of research is dedicated to the synthesis and secretion of neurotrophic factors including nerve growth factor (NGF) and brain-derived neurotrophic factor (BDNF) in order to develop novel therapeutic approaches for the treatment of depressive or neurodegenerative disorders (24-32). Considerable efforts devoted to the discovery of novel neurotrophic factors have shown that glial cell line-derived neurotrophic factor (GDNF), a small protein which promotes the survival of many types of neurons, exerts neuroprotective effects (33-36). GDNF mRNA is widely expressed in the peripheral tissues of developing mamalian and avian neurons (37). The most prominent feature of GDNF is its ability to support the survival of dopaminergic and motor neurons which die in the course of Parkinson’s disease or amyotrophic lateral sclerosis (38-42). According to Hisaoka *et al*, antidepressant drugs increase GDNF release from C6 glioma cells, a rich source of GDNF that may be implicated in their neuroprotective properties (43). This background prompted us to investigate whether progesterone is able to stimulate GDNF secretion from C6 glioma cells. 

## Materials and Methods

***Cell culture and drug treatment***

Rat C6 glioma cell line was obtained from the National Cell Bank of Iran (NCBI, Pasteur Institute of Iran, Tehran, Iran). We used two different culture media: Dulbecco’s Modified Eagle’s Medium (DMEM) and serum-free Opti-MEM (Gibco, UK). All media contained 100 U/ml penicillin and 100 µg/ml streptomycin (Sigma Aldrich, Germany). Incubations were conducted at 37 ^°^C in 5% CO_2_ and 95% air. C6 cells were grown in DMEM supplemented with 2 mM/l L-glutamine and 5% fetal bovine serum (FBS, Gibco). Cells were seeded into 6-well plates at a density of 410^5^/ml in 1 ml of growth medium, allowed to adhere for 24 hr and then medium was replaced with serum-free Opti-MEM containing 0.5% bovine serum albumin (BSA, Gibco). Afterwards, the cells were incubated for 24 hr and the medium was replaced with fresh Opti-MEM and 1 ml of 0.5% BSA containing progesterone (Sigma Aldrich, Germany) at concentrations of 100 nanomolar (nM), 1 micromolar (µM), and 10 µM (7). Cells were treated with progesterone for 12, 24, 36, 48 and 72 hr (43). **Control** cultures **consisted** of the culture **media** and **vehicle** of progesterone (ethanol, Sigma). The concentration of ethanol in the culture was less than 0.01%. At the end of each time period, the conditioned medium was aspirated and the GDNF protein level was measured using a GDNF ELISA kit (Chemicon, UK) according to the manufacturer’s instructions. Briefly, 96-well, flat-bottomed plates were coated with anti-GDNF monoclonal antibody and incubated overnight at 4 ^°^C. Samples and standards were incubated at room temperature for 6 hr. The captured GDNF was incubated overnight at 4 ^°^C with chicken anti-human GDNF polyclonal antibody. After washing the plates, horseradish peroxidise-conjugated anti-chicken immunoglobulin Y antibody was added to the plates (100 μl per well) and incubated at room temperature for 2 hr. The plates were washed and the enzyme substrate was added (100 μl per well). Then, the plates were incubated for 15 min at room temperature in the dark. The absorbance at a wavelength of 450 nm was recorded on a microplate reader (Molecular Devices, UK). In order to evaluate whether the release of GDNF was the result of leakage from damaged cells, we measured the amounts of GDNF located within the C6 cells as well as the effect of progesterone on cell numbers. For the measurement of intracellular GDNF content, cells were lysed with 1% NP-40 cell lysis buffer (Sigma) and GDNF protein level was detrermined by GDNF ELISA kit. Cell numbers were determined using the CCK-8 assay (Cell Counting Kit-8, Roche Diagnostics, Germany). Briefly, after the treatment with different concentrations of progesterone, 10 μl of CCK-8 solution was added to each well in a 96-well plate. After the incubation for 2 hr at 37 ^°^C, the absorbance of the samples at the wavelength of 450 nm was measured by a UV Max kinetic microplate reader (Molecular Devices, UK). In this assay, WST-8-[2-methoxy-4-nitrophenyl]-3-[4-nitrophenyl]-5-[2,4-disulfophenyl]-2H-tetrazolium, monosodium salt) is reduced by dehydrogenases in the cells to give a soluble yellow-coloured product (formazan). The amount of formazan dye generated by the activity of dehydrogenases is directly proportional to the number of living cells.

***Statistics***

The effects of progesterone on GDNF protein levels and C6 glioma cell numbers were analyzed using analysis of variance (ANOVA) followed by Tukey’s *post-hoc* test. Data are expressed as mean±SEM. The level of significance was set at *P*< 0.05. 

## Results

***The effects of progesterone on GDNF content of conditioned media***

**Figure 1:**
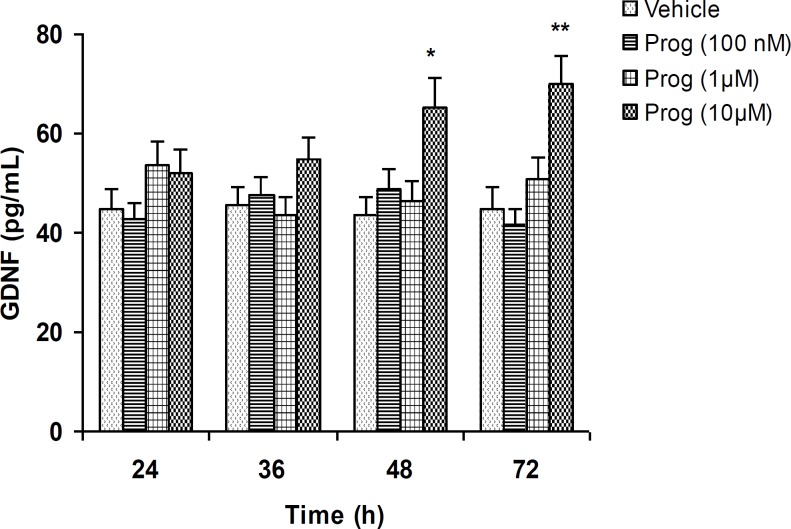
The effects of progesterone on GDNF content in cultured C6 cells. Treatment with progesterone resulted in a significant elevation of GDNF protein level in a concentration- and time-dependent manner.

Forty-eight hr after the treatment with progesterone (10 µM), the secretion of GDNF was significantly increased from the cultured C6 glioma cells into the medium (Figure 1, *P*< 0.05). GDNF protein level remained elevated up to 72 hr (Figure 1, *P*< 0.01). After the treatment for 24 and 36 hr, progesterone did not affect GDNF secretion as compared with vehicle-treated control groups (Figure 1). Lower concentrations of progesterone did not induce any significant change in GDNF protein content (Figure 1). 

***The effects of progesterone on GDNF levels in cell lysates***

**Figure 2:**
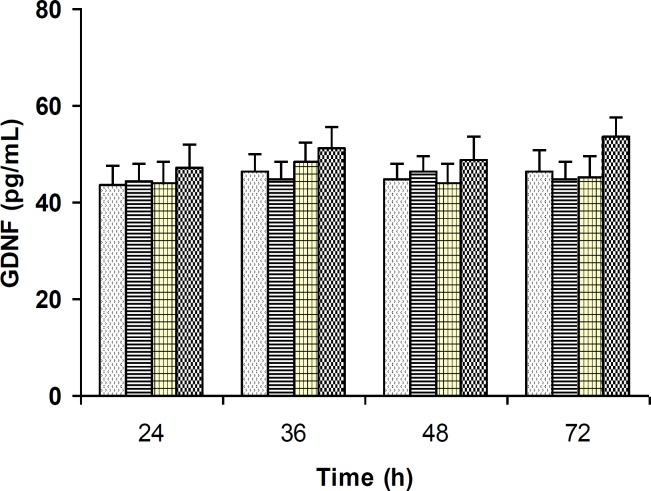
The effects of progesterone on intracellular GDNF content. GDNF level was not altered by treatment with progesterone at any concentration or time point tested. Data are presented as mean±SEM (n=6)

Treating the C6 cells with different concentrations of progesterone for 24, 36, 48, and 72 hr did not alter GDNF protein levels in the cell lysates (Figure 2).

***The effects of progesterone on the cell growth of C6 cells***

**Figure 3:**
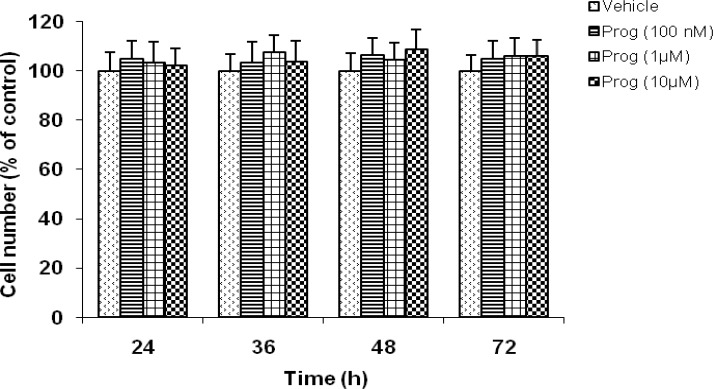
The effects of progesterone on the cell growth of C6 cells. Treating the cells with progesterone at concentrations ranging from 100 nM to 10 µM for up to 72 h in the serum-free conditions had no effect on cell numbers. Data are presented as mean±SEM (n=6)

Treating the C6 cells with progesterone at concentrations ranging from 100 nM to 10 µM in the serum-free conditions had no effect on cell numbers at any time point tested (Figure 3).

Data are presented as mean±SEM (n=6). *P<0.05, **P< 0.01 vs. vehicle group.

(nM: nanomolar, µM: micromolar, Prog: progesterone)

## Discussion

Progesterone, in addition to its effects on the reproductive system, has been shown to exert beneficial and neuroprotective effects in the injured central and peripheral nervous systems. There is considerable evidence that progesterone limits tissue damage and improves functional outcome after traumatic brain injury, stroke, spinal cord injury, diabetic neuropathies, and other types of acute neuroinjury in several species (14, 44-50). Meanwhile, the probable mechanisms underlying the neuroprotective effects of progesterone still remain elusive. In the present work, we evaluated the effects of progesterone on GDNF secretion from C6 glioma cells as an in vitro model system. As it is observed in Figure 1, progesterone significantly elevated GDNF release in concentration- and time-dependent fashion. In parallel, we measured the amounts of GDNF located within the C6 cells in order to evaluate whether the release of GDNF was the result of the leakage from damaged cells. We found that treating the C6 cells with various concentrations of progesterone for up to 72 h did not alter the amount of GDNF present in the cell lysates (Figure 2). Furthermore, treating the cells with progesterone in the serum-free conditions had no effect on the cell numbers (Figure 3), indicating that progesterone had no effect on cell proliferation or cell death. Therefore, these findings indicate that progesterone-induced elevation of GDNF content in the conditioned media is not due to the leakage from damaged cells. If GDNF secretion was simply a consequence of leakage from the cells damaged by progesterone treatment, therefore a decrease in intracellular GDNF levels or the number of cells might have been observed over time, but such significant decreases were not found following progesterone treatment (Figures 2 and 3). In addition, if progesterone-induced secretion of GDNF was due to the leakage of GDNF from damaged cells, a continuous release might have been expected over time, but the time course of GDNF release showed that a significant release was observed only after a 48 hr incubation period but not at earlier time points (Figure 1). Therefore, it appears that enhancement of GDNF secretion by glial cells is implicated, at least in part, in the mechanism of action of progesterone that, in turn, may result in neuroprotection and restoration of neuronal integrity and plasticity. As aforementioned, the probable mechanisms underlying the neuroprotective effects of progesterone have not yet been fully understood, as progesterone does not target a single class of receptors or one cell type (51). In fact, progesterone has manifold actions in the brain, therefore, this neuroactive steroid may alter the expression of as-yet-unidentified gene(s) and proteins involved in the cytotoxic or repair processes. As previously reported, prevention of inflammation (52), excitotoxicity (53), and apoptosis (54, 55), as well as promoting remyelinization (56, 57) may be implicated in the neuroprotective action of progesterone. According to Kaur *et al*, progesterone may exert protective effects through its ability to elicit activation of specific signaling pathways relevant to neuroprotection (58). Regarding the effects of progesterone on neurotrophic factors, progesterone-induced upregulation of BDNF expression has previously been reported (58, 59). Our findings demonstrate that progesterone is able to elevate GDNF secretion from C6 glioma cells (Figure 1). This offers a novel mechanism through which progesterone may exert its neuroprotective effects. In addition, since glial cells are implicated in the development, survival, and metabolism of neuronal cells (60) and modulate the synaptogenesis in the brain (61-63), therefore, progesterone by *in vivo *stimulation of GDNF secretion from glial cells may be proved to be beneficial for neurodegenerative disorders such as Alzheimer’s disease.

## Conclusions

Progesterone is able to increase GDNF secretion from glial cells. This, represents a new pathway through which progesterone may protect neurons and restore neuronal integrity and plasticity.
